# Genome-wide identification, characterization and expression analysis of *BES1* gene family in tomato

**DOI:** 10.1186/s12870-021-02933-7

**Published:** 2021-03-30

**Authors:** Deding Su, Wei Xiang, Ling Wen, Wang Lu, Yuan Shi, Yudong Liu, Zhengguo Li

**Affiliations:** 1 Key Laboratory of Plant Hormones and Development Regulation of Chongqing, School of Life Sciences, Chongqing University, Chongqing, 401331 China; 2 Center of Plant Functional Genomics, Institute of Advanced Interdisciplinary Studies, Chongqing University, Chongqing, 401331 China

**Keywords:** *BES1* gene family, Genome-wide analysis, Subcellular localization, Transactivation activity, Expression pattern, Tomato (*Solanum lycopersicum*)

## Abstract

**Background:**

As the key regulators in BR signaling, *BES1* family genes regulate thousands of target genes involved in various development processes. So far, the functions of *BES1* family are poorly understood in tomato, and a comprehensive genomic and expressional analysis is worth to conduct for this family.

**Results:**

Here, nine *SlBES1* family members were identified in tomato and classified into five groups based on the conserved motif, gene structure and phylogenetic analysis. Synteny among tomato, *Arabidopsis*, pepper and rice were further analyzed to obtain insights into evolutionary characteristics. Several *cis*-elements related to hormone, stress and plant development were exhibited in the promoter regions of *SlBES1* family genes. Subcellular localization showed seven members localized both in the nucleus and cytoplasm, implying the presence of dephosphorylated and phosphorylated form of these seven proteins, furthermore, five of them possessed transcription activation activity whereas the left two functioned as transcriptional repressors. Another two members, however, neither localized in the nucleus nor had transactivation activity. Besides, *SlBES1.8* showed flower-specific expression while other members expressed ubiquitously in all organs. Moreover, *SlBES1* genes exhibited variational expression in response to nine principal plant hormones. Notably, the expression levels of *SlBES1* genes presented a dominant downregulated trend in response to stresses.

**Conclusions:**

In this study, we systematically analyzed the genomic characterization of *SlBES1* family, together with the analyses of protein functional features and expression patterns, our results lay a foundation for the functional research of *SlBES1* family.

**Supplementary Information:**

The online version contains supplementary material available at 10.1186/s12870-021-02933-7.

## Background

Plant growth and development are continuously regulated by the integration of plant hormones. Meanwhile, their yield and quality are often influenced by various of environmental stimuli including biotic and abiotic stresses. To adapt the adverse environment, many genes especially for those transcription factors (TFs) tend to integrate multiple hormone signaling to against the environmental stimuli and maintain normal growth and development [1, 2].

It’s well known that plant hormones contain but not limited to auxin, cytokinin (CK), gibberellin (GA), abscisic acid (ABA), ethylene (ET), brassinosteroid (BR), salicylic acid (SA), jasmonic acid (JA) and strigolactone. Among them, since the discovery in *Brassica napus* pollen, BR has been regarded as a plant growth-promoting hormone for its effects on cell elongation and division [3, 4]. Subsequent studies indicate that BRs play multifunctional roles in plant developmental and physiological processes, including seed germination, plant architecture, vascular differentiation, stomata formation, flowering, male fertility, senescence, and stress resistance [5–7]. Meanwhile, the BR signal transduction pathway has been extensively explored by using genetic, molecular, and proteomic approaches over the past few decades, providing us a global view on the molecular mechanism of BR function. Briefly, an extracellular leucine-rich repeat receptor kinase (LRR-RK), BRASSINOSTEROID-INSENSITIVE 1 (BRI1), can recognize and bind the BR, leading to the initiation of BR signaling [8, 9]. A series of kinases and phosphatases in the pathway are successively triggered to fulfil their functions, resulting the dephosphorylation and activation of two homologous transcription factors, BRI1-EMS-SUPPRESSOR 1 (BES1) and BRASSINAZOLE-RESISTANT 1 (BZR1) [10–13], which in turn regulate thousands of target genes by binding to the E-box (CANNTG) or BR-response element (BRRE, CGTGT/CG) [14, 15].

The roles of BES1 and BZR1 in BR signal pathway are first illuminated by two dominant mutations, *bes1-D* and *bzr1-D*, which exhibit constitutive BR response phenotypes including suppressed *bri1* dwarf phenotype, insensitivity to brassinazole (BRZ), elongated stem, accelerated senescence and upregulated expression of BR-induced genes [12, 13]. Since then, the vitally important functions of BES1/BZR1 in integrating multiple hormone signaling to regulate plant growth and development are widely explored. For example, BES1/BZR1 can directly regulate the expression of *CELLULOSE SYNTHASE GENES* (*CESAs*) [16], *MICROTUBULE DESTABILIZING PROTEIN 40* (*MDP40*) [17], *ATBS1-INTERACTING FACTOR 2* (*AIF2*) [18], *INCREASED LEAF INCLINATION 1* (*ILI1*), *PACLOBUTRAZOL RESISTANCE 1* (*PRE1*) and *ILI1 BINDING bHLH* (*IBH1*) [19] or interact with MYB DOMAIN PROTEIN 30 (AtMYB30) [20], MYELOBLASTOSIS FAMILY TRANSCRIPTION FACTOR-LIKE 2 (MYBL2) [21] and HETEROTRIMERIC G-PROTEIN β SUBUNIT (AGB1) [22] to control plant cell elongation. Analogously, BES1/BZR1 can also influence plant immunity, stress responses, floral organ development and cell division and differentiation in quiescent center by directly regulating the expression of related key genes or interacting with relevant proteins [7]. A large number of putative target genes have been identified by chromatin immunoprecipitation-microarray (ChIP-chip) studies for BES1 and BZR1, up to 1609 and 3410 respectively. Among which numerous genes are under the regulation of BRs, while there are a number of target genes involved in other signaling like plant hormone and stress signaling [23, 24], implying that BES1 and BZR1 are not only the master regulators in BR signaling but also play critical roles in other regulatory networks.

There are four *BES1* homologs in *A. thaliana*, named *BEH1–4*. However, few studies are focus on these four genes individually, probably because their functional redundancy in BR signaling [15]. In *Arabidopsis*, individual single mutant of *BES1*, *BZR1* and their four homologs didn’t show any growth defects. Moreover, no obvious phenotypes were observed from those different combinations of double, triple, and quadruple mutants. While the male sterility phenotype, tapetum and microsporocyte developmental defects, was exhibited in quintuple mutant (*bes1 bzr1 beh1 beh3 beh4*) and sextuple mutant (*bes1 bzr1 beh1 beh2 beh3 beh4*) [25]. Meanwhile, the similar results were demonstrated by another study reported by Chen et al. [26], indicating the highly functional redundancy of *BES1* genes. The left two BES1 members, BAM7 and BAM8 (also named BMY4 and BMY2 correspondingly), are β-amylase proteins but included in *BES1* family for the presence of BES1-type domain in the N-terminal. Interestingly, these two β-amylases are reported to function as TF and function in controlling shoot growth and development by mediating BR signaling in *Arabidopsis* [27].

In a word, *BES1* family genes act as key regulators in plant growth and development by orchestrating BR signaling and other signal pathways. However, understandings of *BES1* gene family are mainly based on the studies performed in *Arabidopsis*, thus it’s essential to obtain fresh insights from other plant species particularly from crops. As one of the most important horticultural crops, tomato (*Solanum lycopersicum*) is a typical model for the research of plant growth and development especially for the fleshy-fruit development and ripening [28]. In our study, a comprehensive genome-wide analysis of *SlBES1* gene family was performed, including their chromosomal distribution, conserved amino acid residues within the BES1-type domain, phylogenetic relationship, synteny analysis, gene structure, conserved motifs and potential *cis*-elements. We further explored their subcellular localization and transcriptional activation activity. What’s more, the spatio-temporal expression patterns of *SlBES1* gene family were also investigated. More important, we detailedly analyzed the responsiveness of *SlBES1* gene family to the nine principal plant hormones and to different stresses. Our results provide valuable information to the functional and mechanism analysis of *BES1* family genes in tomato. Moreover, this study may lay a foundation for the research of plant hormone signaling and stress resistance.

## Results

### Identification and characterization of *SlBES1* gene family

To identify *BES1* gene family in tomato, the conserved BES1-type domain sequence collected from AtBES1 was used as a BLASTP query in Solanaceae Genomics Database. Totally 9 putative *SlBES1* candidates were obtained with default parameters. Meanwhile, Phytozome database was also used to search *SlBES1* gene family, and the same *SlBES1* candidates were obtained. Then the presence of conserved BES1-type domain was confirmed by CD-Search and SMART. These 9 *SlBES1* genes was subsequently named as *SlBES1.1* to *SlBES1.9* according to their genomic locus (Table 1). Particularly, two members (*SlBES1.1* and *SlBES1.7*) were annotated as β-amylases whereas other were annotated as TFs. The annotated seven *SlBES1* TFs showed relative less exon number ranged from 2 to 3, shorted protein length ranged from 180 to 333 amino acids (AA) and lighter predicted molecular weight ranged from 20,389 to 35,772.85 kDa. While those two annotated β-amylases had more exon number, longer protein length and bigger molecular weight (Table 1). Other detailed properties of *SlBES1* genes like theoretical isoelectric point (pI) and BES1-type domain position were also provided in Table 1.

**Table 1:** Characteristics of *SlBES1* genes and the encoded proteins identified in tomato

Gene name	Gene accession No.	Genomic locus	Exon number	AA^a^	MW^b^ (kDa)	pI^c^	BES1-type domains position
SlBES1.1	Solyc01g094580	SL2.50ch01:85997496..86006359	11	695	77,864.45	5.37	70–204
SlBES1.2	Solyc02g063010	SL2.50ch02:35030416..35032639	2	319	34,474.87	9.38	38–119
SlBES1.3	Solyc02g071990	SL2.50ch02:41313401..41318179	3	324	34,908.89	8.14	31–130
SlBES1.4	Solyc03g005990	SL2.50ch03:667344..672399	3	323	34,696.65	8.18	31–132
SlBES1.5	Solyc04g079980	SL2.50ch04:64289859..64291884	2	328	35,108.38	8.88	52–139
SlBES1.6	Solyc07g062260	SL2.50ch07:65038606..65041740	3	315	33,827.99	9	3–99
SlBES1.7	Solyc08g005780	SL2.50ch08:604998..612717	10	666	75,255.45	6.09	72–202
SlBES1.8	Solyc10g076390	SL2.50ch10:59363764..59364788	2	180	20,389	8.68	37–122
SlBES1.9	Solyc12g089040	SL2.50ch12:64193208..64195373	2	333	35,772.85	8.85	59–145

^a^AA Number of amino acids; ^b^MW Molecular weight; ^c^*pI* Theoretical Isoelectric point

### Chromosomal distribution and conserved amino acid residues analysis of *SlBES1* genes

**Fig. 1:**
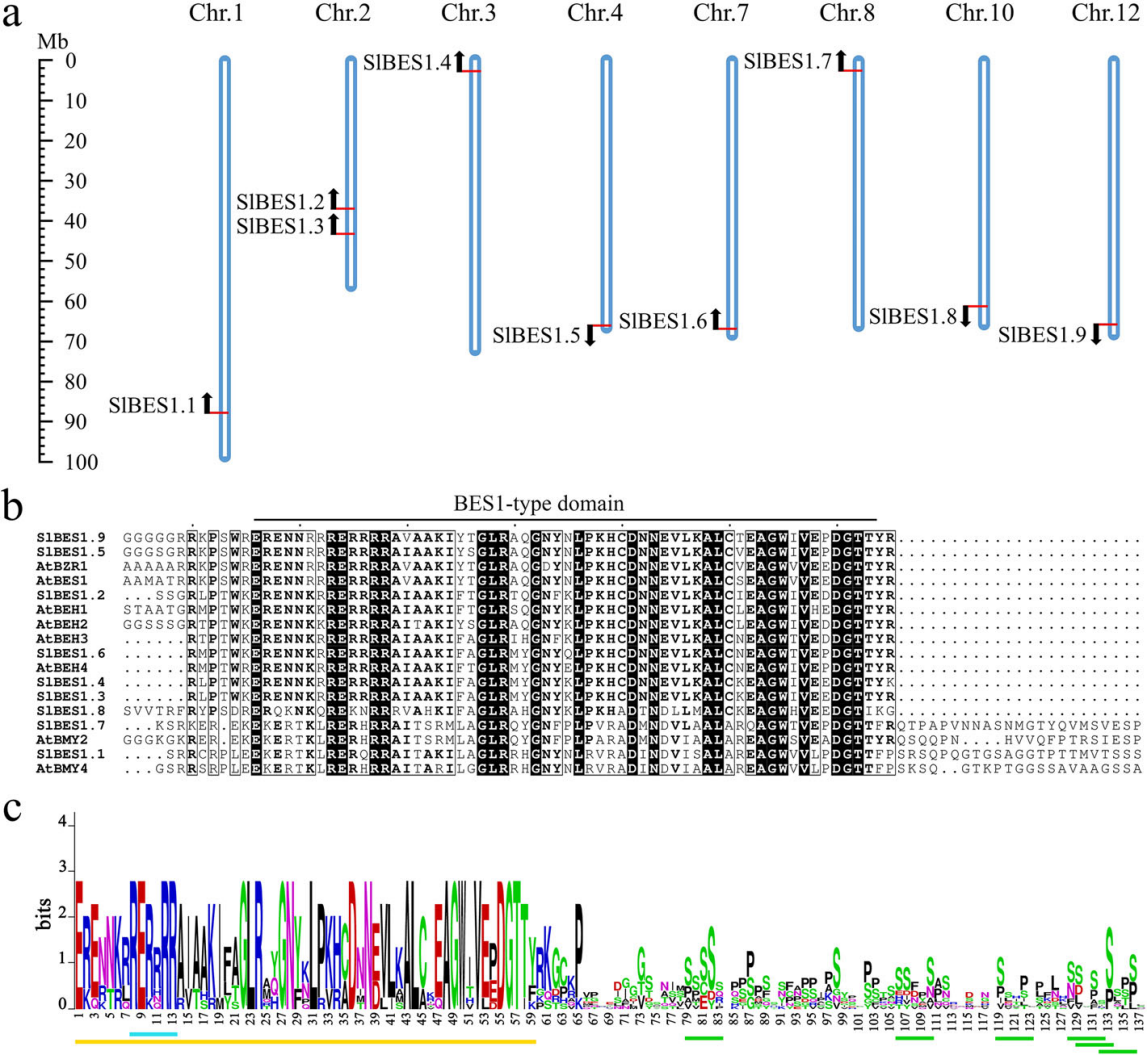
Chromosomal distribution and conserved protein sequence analysis of *SlBES1* genes. **a** Chromosomal distribution of the nine *SlBES1* genes on tomato genome. Red line and arrow indicate the position and the direction of *SlBES1* genes respectively. **b** Multiple sequence alignment of BES1-type domain (marked with underline) of tomato and *Arabidopsis* BES1 proteins by ClustalX. **c** Sequence logos of highly conserved amino acid residues of BES1-type domain in tomato. Yellow line indicates the conserved N-terminal BES1-type domain, blue line indicates the arginine composition biases, green lines indicate the serine-rich phosphorylation sites

*SlBES1* gene family distributed on 8 chromosomes randomly, each *SlBES1* gene located at one independent chromosome except chr.2 containing two *SlBES1* genes, *SlBES1.2* and *SlBES1.3*. Notably, most of *SlBES1* genes positioned on distal ends of chromosomes, three of them distributed in a forward direction, while other six members distributed in a reverse direction (Fig. 1a).

The length of BES1-type domain was 86 to 135 amino acids in tomato. From the alignment of full length sequences, the comparative conserved sequences only showed in the N-terminal of BES1-type domain (Fig. 1b). We further analyzed the conservation of amino acids residues in this domain, similar to the analysis in *A. thaliana*, *O. sativa* and *G. hirsutum* [29], the amino acids residues in the N-terminal BES1-type domain remained conserved at most of loci, which was assumed to be required for DNA binding. Remarkably, an arginine bias region between amino acids 8 to 13 was also observed in *SlBES1* family. The C-terminal sequence of BES1-type domain was less conserved, it harbored many serine-rich phosphorylation sites in contrast, which implied the potentially regulatory center of SlBES1 proteins (Fig. 1c) [30].

### Phylogenetic and Syntenic analysis of *SlBES1* genes

**Fig. 2:**
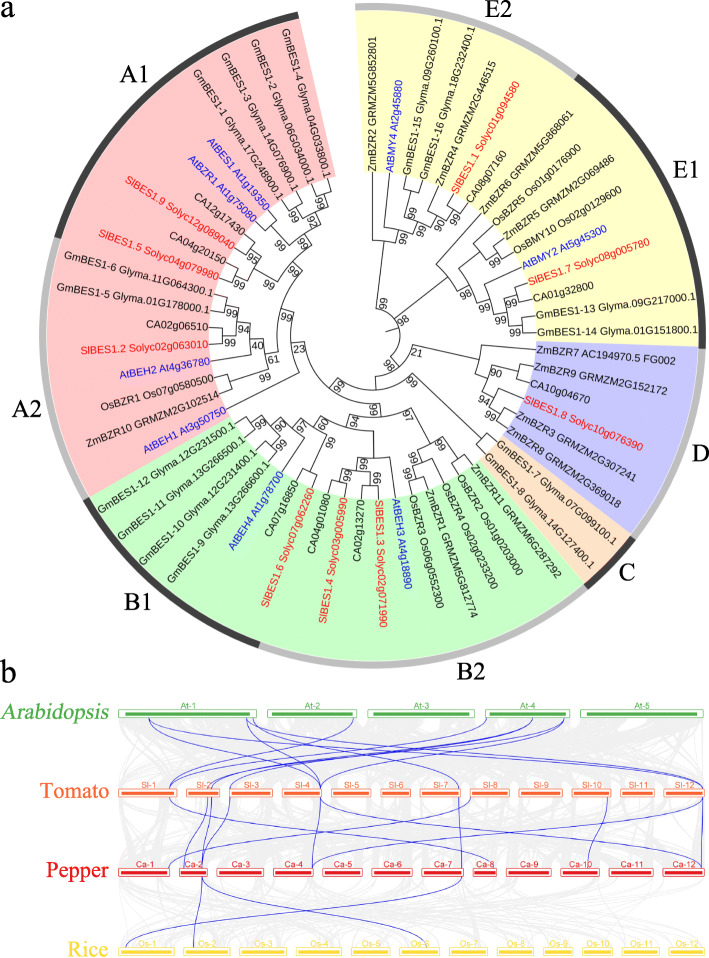
Phylogenetic analysis of *SlBES1* family. **a** Phylogenetic tree of BES1 proteins from tomato and other plants. The phylogenetic tree was constructed by MEGA X according to NJ method. 9, 8, 9, 16, 6 and 11 BES1 protein sequences from *S. lycopersicum* (red font), *A. thaliana* (blue font), *C. annuum*, *G. max*, *O. sativa* and *Z. mays* respectively were used. Group A-E are distinguished by different colors. Bootstrap values are provided near nodes. **b** Synteny analysis of *SlBES1* genes among tomato, pepper, *Arabidopsis* and rice. The gray lines indicated the collinear blocks within these four species genomes, and the syntenic *BES1* gene pairs were highlighted with the blue lines

To understand the phylogenetic relationship of *SlBES1* family genes, a total of 59 *BES1* genes from *S. lycopersicum* (9), *A. thaliana* (8), *C. annuum* (9), *G. max* (16), *O. sativa* (6) and *Z. mays* (11), were used to construct Neighbor-Joining phylogenetic tree by MEGA X with default parameters. In keeping with the trees conducted by Liu et al. [29], Li et al. [31] and Song et al. [32], we grouped these 59 *BES1* genes into five groups, named A to E, based on the bootstrap values and phylogenetic topology (Fig. 2a). Group A, B and E possessed the majority of *BES1* genes and were further divided into 2 subgroups respectively. Subgroup A1 contained the key members *BES1* and *BZR1*, which were the homologs of *SlBES1.5* and *SlBES1.9* respectively in tomato. As analyzed by Liu et al. [29], the corresponding group E was more ancient than other groups, and it was true that this group harbored *BES1* genes from all of six species analyzed here. Additionally, genes in group E showed quite longer amino acids length and were annotated as β-amylases discriminatively. Group D contained quite less *BES1* genes from three species, including one tomato *BES1* gene, *SlBES1.8*, and group C specifically possessed two *BES1* genes from *G. max*, this result showed the expansion and divergence of *BES1* gene family in evolution.

To further understand the phylogenetic mechanisms of *SlBES1* family, a comparative syntenic maps was conducted among three dicots (tomato, pepper and *Arabidopsis*) and one monocot (rice) (Fig. 2b). The results showed that the most tomato *BES1* homologs presented in pepper, another *solanaceae* species, followed by *Arabidopsis*, and the monocot rice exhibited the fewest homologs. What’s more, all *SlBES1* syntenic genes (9) could be found on pepper chromosome, and most of *SlBES1* syntenic genes (7) could be found on *Arabidopsis* chromosome, while only two exhibited on rice chromosome. Taken together, the syntenic gene pairs of *SlBES1* were more presented in dicot than in monocot. Meanwhile, as the *solanaceae* relative of tomato, pepper possessed superior synteny with tomato than *Arabidopsis* and rice. These results suggested that *BES1* family may play important roles to plant evolution.

### Gene structure and amino acids conserved motif of *SlBES1* genes

**Fig. 3:**
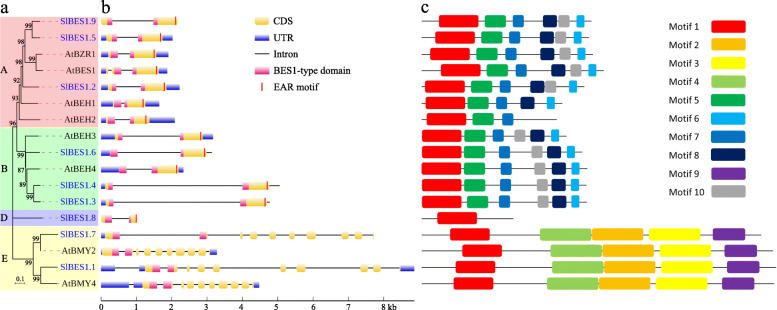
The gene structures and conserved motifs of *SlBES1* and *AtBES1* genes. **a** The NJ-tree constructed with BES1 proteins from tomato (blue font) and *Arabidopsis*. **b** The structures of *SlBES1* and *AtBES1* genes visualized by GSDS 2.0. The coding sequence (CDS), untranslated region (UTR), BES1-type domain and EAR motif are displayed in different colors, and the lines between boxes represent introns. **c** Conserved domains of SlBES1 and AtBES1 proteins analyzed by MEME suite. Different color boxes indicate different kinds of motifs

With the evolution, genes tend to diverge their regulatory and/or coding regions based on the gene duplication. Thus amino acid-altering substitutions and/or alterations may occur, and function of genes could be changed to adapt different growth conditions [33]. A simpler Neighbor-Joining phylogenetic tree was constructed by using BES1 protein sequences from *S. lycopersicum* and *A. thaliana* to fully analyze the gene structure and conserved motif (Fig. 3a).

Structures of *BES1* genes clustered in the same clade were very close, including number and position of exons and introns. For example, the annotated β-amylase genes contained much more exons (10 to 11) than those annotated TFs that obtained only 2 to 3 exons generally, and the third exon of those three tomato *BES1* genes (*SlBES1.3*, *SlBES1.4* and *SlBES1.6*) had only 4 nucleotides. Furthermore, most of introns of tomato *BES1* genes appeared to be longer than their *Arabidopsis* homologs, which agreed with the fact that tomato had the bigger genome. Besides, the BES1-type domain of tomato *BES1* genes was all located between exon1 and exon2 except *SlBES1.1*. Noticeably, a LxLxL type ethylene-responsive element binding factor-associated amphiphilic repression (EAR) motif, which was previously reported as a negative transcriptional regulatory motif [34], was observed in the C-terminal end of those *BES1* genes annotated as TFs, implying a potential transcriptional inhibition function of these *BES1* genes, while those annotated β-amylase genes didn’t contain this special motif (Fig. 3b).

Proteins containing highly consistent amino acid sequences particularly in functional domain tended to share similar biological functions, thus 10 conserved motifs of tomato and *Arabidopsis* BES1 proteins were explored by the MEME suite (Fig. S1). As shown in Fig. 3c, motif 1 was the most conserved motif exhibited in all BES1 proteins and it overlapped with BES1-type domain. The permutation and combination of these motifs were very closely related with their phylogenetic relationship. For example, group A and B shared the same motifs (motif 1, 5, 6, 7, 8 and 10) while exhibited an opposite order between motif 8 and 10, and the rest of motifs (motif 1, 2, 3, 4 and 9) were included into group E. Specially, group D exclusively contained the motif 1, suggesting a potential loss of function or functional differentiation of gene in this group.

### Potential *cis*-element in *SlBES1* gene promoters

**Fig. 4:**
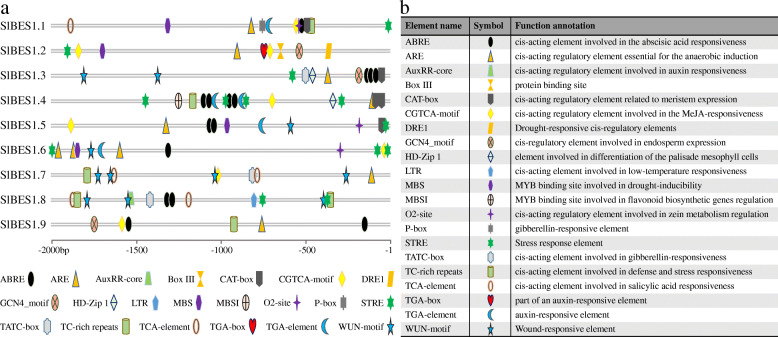
*Cis*-element analysis in the promoters of *SlBES1* genes. **a** Locations of *cis*-elements in the 2 kb sequences upstream of *SlBES1* genes. Different kinds of *cis*-elements are represented with different symbols. **b** The detailed functional annotations of *cis*-elements

To explore the potential *cis*-elements, 2 kb upstream sequence of *SlBES1* genes were submitted to PlantCARE database. The kind and position of *cis*-elements were marked as different icons (Fig. 4a), and their potential functions were annotated in Fig. 4b. All of these *cis*-elements detected could be mainly classified into three types: phytohormone responsive, plant development-related and stress responsive elements. Among these *cis*-elements, ABRE and STRE were conspicuous, which were involved in abscisic acid and stress responsiveness respectively, indicating that *SlBES1* genes may be able to be induced or repressed by abiotic stress and subsequently participate in plant stress resistance. Besides, each of *SlBES1* gene possessed different kinds and amount of *cis*-elements, we may assume that, under different growing status and environmental conditions, *SlBES1* genes could function independently or synergistically to ensure plant normal growth and development.

### Subcellular localization of SlBES1 proteins

**Fig. 5:**
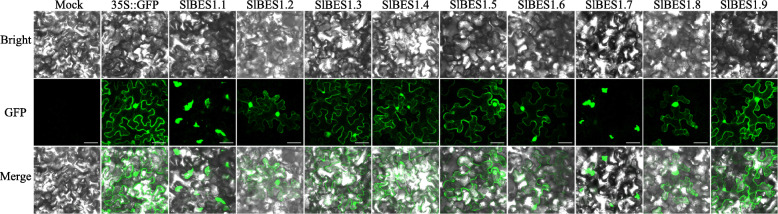
Subcellular localization analysis of SlBES1 proteins. Tobacco (*Nicotiana benthamiana*) leaves transiently expressed SlBES1-GFP fusion proteins were observed through the laser scanning confocal microscope. Scale bars represent 50 μm

Subcellular localization implied the working position of a protein and was nonnegligible for gene functional research. To detect the subcellular localization of SlBES1 proteins, green fluorescent protein (GFP) fused with SlBES1 proteins was used to transiently express in tobacco (*Nicotiana benthamiana*) leaf. As shown in Fig. 5, seven BES1 proteins, SlBES1.2, SlBES1.3, SlBES1.4, SlBES1.5, SlBES1.6, SlBES1.8 and SlBES1.9, localized both in the nucleus and cytoplasm. This result was basically consistent with the fact that phosphorylated BES1 mainly distributed in the cytoplasm while dephosphorylated BES1 accumulated in the nucleus [15]. For those two annotated β-amylase proteins, SlBES1.1 and SlBES1.7, the green fluorescence pigment showed a non-nuclear shape, thus we further used DAPI to mark the nucleus of tobacco leaf cell, and the green fluorescence pigment was truly not overlapped with the nucleus (Fig. S2). The chlorophyll auto-fluorescence was also detected to analyze if these two proteins localized to the chloroplast, while clear distinction was observed between these two fluorescence pigments both in the size and position, indicating a non-chloroplastic localization (Fig. S2). Taken together, given the bigger size of the green fluorescence pigment than the nucleus, we assumed that these two annotated β-amylase genes localized in the endoplasmic reticulum.

### Transactivation activity analysis of SlBES1 proteins

**Fig. 6:**
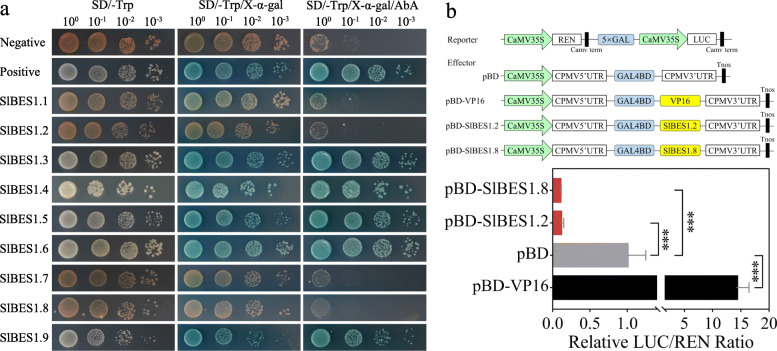
Transactivation activity analysis of SlBES1 proteins. **a** Transactivation activity analysis of SlBES1 proteins in yeast. The pGBKT7-SlBES1 fusion vectors were transformed into Y2H Gold yeast cells. The transformants were cultivated on SD/−Trp medium and screened by X-α-Gal and Aureobasidin A (AbA). **b** Transcriptional repression activity analysis of *SlBES1.2* and *SlBES1.8* by dual-luciferase assay. The vector construction of effector and reporters are shown above. VP16 and pBD alone were used as positive and negative control respectively. The ratio of LUC to REN indicates the trans-repression ability of SlBES1.2 and SlBES1.8. Value of each column represents the mean ± SE of six biological replicates. *** refer to significant differences with *p* < 0.001

As most of *SlBES1* genes were annotated as TFs, the transcriptional activation activity was necessary to be analyzed, hence the GAL4-responsive reporter system in yeast was used to detect the transactivation activity of SlBES1 proteins (Fig. 6a). After transformed the pGBKT7-SlBES1 fusion plasmids into yeast for 3 days, all yeast transformants grew well on SD/−Trp medium, while only those five yeast transformants containing pGBKT7-SlBES1.3, pGBKT7-SlBES1.4, pGBKT7-SlBES1.5, pGBKT7-SlBES1.6, pGBKT7-SlBES1.9 respectively and positive yeast transformant hydrolyzed X-α-Gal and showed the blue pigment and survived from Aureobasidin A (AbA) screening, indicating that these five SlBES1s had transactivation activity whereas other four SlBES1s, including SlBES1.1, SlBES1.2, SlBES1.7 and SlBES1.8, had no transactivation activity. According to the non-nuclear subcellular localization of SlBES1.1 and SlBES1.7 presented above, we assumed that these two SlBES1 proteins were not TFs (Fig. 5), consistent with this assumption, SlBES1.1 and SlBES1.7 truly didn’t have the transactivation activity (Fig. 6a). However, given the presence of EAR motif in the C-terminal end of those seven SlBES1 TFs (Fig. 3b), it was unexpected that five of them discovered to possess the transactivation activity (further discussed in Discussion). The rest of two SlBES1 proteins, SlBES1.2 and SlBES1.8, showing had no transactivation activity, were further to be ascertained if they acted as transcriptional repressor by dual-luciferase assay (Fig. 6b). Full length of coding sequences of these two genes were fused with GAL4 DNA binding domain as the effector. A strong transcriptional activator, VP16 [35], was used as a positive control. After co-expressed of effector and reporter in tobacco leaf, the LUC and REN value was measured. As anticipated, the relative LUC/REN ratios of pBD-SlBES1.2 and pBD-SlBES1.8 were pretty lower than the pBD alone. As a contrast, the VP16 transcriptional activator significantly increased the expression of the LUC reporter. Together with the transactivation activity analysis in yeast, we confirmed that SlBES1.2 and SlBES1.8 acted as the transcriptional repressor.

### Tissue-specific and spatio-temporal expression profiles of *SlBES1* genes

**Fig. 7:**
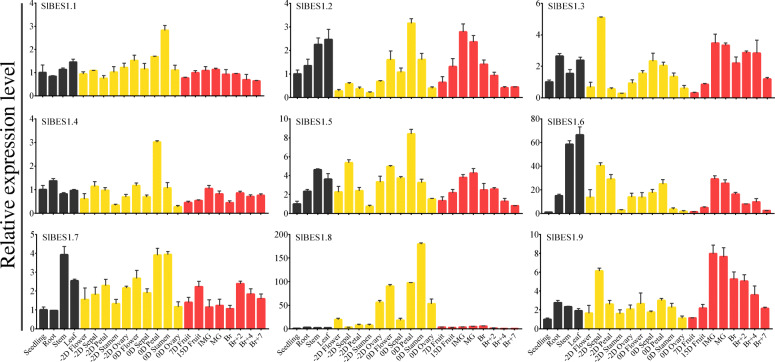
Expression profiles of *SlBES1* genes in different tissues at different developmental stages. Twenty two tissues at different developmental stages were used as the templates. -2D represents 2 days before anthesis. 0D, 7D and 15D represent 0, 7 and 15 days after anthesis respectively. IMG, immature green. MG, mature green. Br, breaker. Br + 2/4/7, 2/4/7 days after Br. The expression level of *SlBES1* genes in seedling was normalized to 1. Value of each column represents the mean ± SE of three biological replicates

Development- and tissue-specific expression pattern could lead us to predict the potential function of a gene, thus the spatio-temporal expressions of *SlBES1* genes were explored by quantitative real-time polymerase chain reaction (qRT-PCR). Twenty two templates of tomato tissue were selected for expression profile detection, including seedling at 12 days post germination (DPG), root, stem, leaf at 30 DPG, flower and floral organ (sepal, petal, stamen and ovary) at anthesis and 2 days before anthesis and fruit at different development stages (7 days and 15 days after anthesis, immature green, mature green, breaker, 2 days, 4 days and 7 days after breaker). In general, most of *SlBES1* genes expressed ubiquitously in all organs detected except *SlBES1.8* that principally expressed in flower organ, indicating a potential important function of *SlBES1.8* during fruit set. Notably, *SlBES1.1* and *SlBES1.4* had the relative stable expression pattern, only a relative higher expression was observed in anthesis stamen and petal respectively, suggesting that *SlBES1.1* and *SlBES1.4* may function fundamentally to tomato plant development. What’s more, the expressions of *SlBES1.2*, *SlBES1.5*, *SlBES1.6* and *SlBES1.9* gradually increased with the development of fruit, reaching the highest level at IMG and MG stages, then decreased gradually with the fruit ripening (Fig. 7). Interestingly, these four genes possessed more close evolutionary relationship than other *SlBES1* members (Fig. 3a), implying a potential functional redundancy or synergistic effect of these four *SlBES1* genes to tomato fruit development.

### Expression profiles of *SlBES1* genes in response to plant hormone

It has been widely studied in the past century that plant hormones played vitally important roles in the regulation of plant growth and development. Understanding of the responsiveness of a gene to plant hormone especially for those TFs could provide us the clue in the research of gene function. In this study, nine major kinds of plant hormones or their analogues, including indole-3-acetic acid (IAA), 6-Benzylaminopurine (6-BA), Gibberellin A3 (GA_3_), Abscisic Acid (ABA), ethephon, epi-brassinolide (EBL), salicylic acid (SA), methyl jasmonate (MeJA) and strigolactone (GR24), were used to treat the tomato seedling at 12 DPG.

First of all, the efficient effects of plant hormone treatment were validated by the reference genes that were reported previously had responsiveness to plant hormone (Fig. S3). *ARF5* [36], *TAS14* [37], *E4* [38], *PR1* [39], *WRKY37* [40] and *D27* [41] could be induced by IAA, ABA, Ethephon, SA, MeJA and GR24 respectively, while *CLAU* [42], *GA20ox1* [43] and *CPD* [44] could be repressed by 6-BA, GA_3_ and EBL respectively. And expectedly, the expression of these genes under corresponding hormone treatment were basically in line with the reports published before, for example, *TAS14* and *PR1* were greatly induced over hundreds times by ABA and SA respectively, suggesting the effective treatment of plant hormone.

**Fig. 8:**
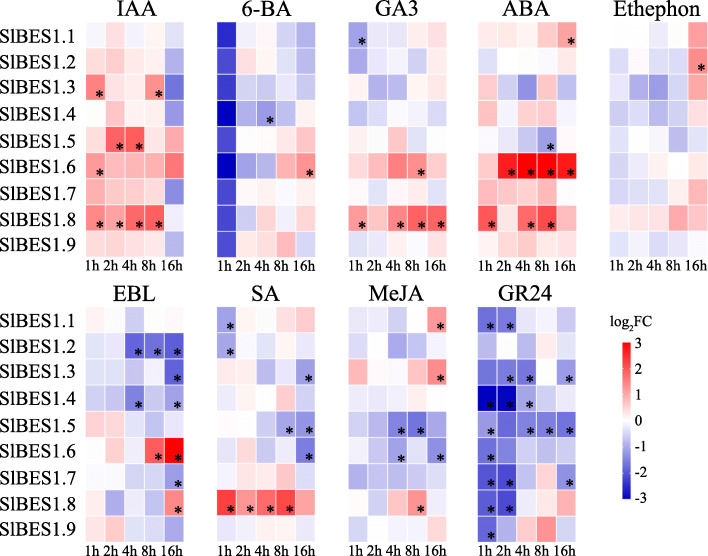
Expression profiles of SlBES1 genes under hormone treatments. Nine principal plant hormones or their analogues including IAA, GA_3_, 6-BA, ABA, Ethephon, EBL, SA, MeJA and GR24 were selected to treat the tomato seedlings at 12 DPG to analyze the responsiveness of *SlBES1* genes to auxin, gibberellin, cytokinin, abscisic acid, ethylene, brassinosteroid, salicylic acid, jasmonic acid and strigolactone respectively. Samples collected from those seedlings treated with liquid MS/2 medium without any plant hormone were used as control. The relative expressions of *SlBES1* genes were detected by qRT-PCR after treated for 1 h, 2 h, 4 h, 8 h and 16 h. Data were converted to log_2_FC (FC, fold change) and visualized by heat map. Value for each time point represents the mean of three biological replicates. Red and blue colors indicate increased and decreased expression levels to the control respectively. * refer to significant differences with *p* < 0.05 compared to the corresponding mock controls

The responsiveness of *SlBES1* genes to these hormones was investigated by qRT-PCR (Fig. 8). The fold change of expression level more than 2 times with *p* value lower than 0.05 (FC > 2, *p* < 0.05) was regarded as having responsiveness to the plant hormone. According to this, the responsiveness of *SlBES1* genes to these nine kinds of plant hormones was summarized in Table S1 and the presence of responsiveness was marked as “Y”. In general, all *SlBES1* genes could response to at least one kind of plant hormone, while the responsiveness to different plant hormone was distinguishing. For example, *SlBES1.6* could response to 8 kinds of plant hormones while *SlBES1.9* could only response to one, i.e. GR24. On the other hand, GR24 could affect the maximum number of *SlBES1* gene, up to 8 members, indicating that *SlBES1* genes may have potential connection with strigolactone signaling. However, ethephon could only influence the expression of *SlBES1.2*. Besides, *SlBES1* genes showed an identical trend in response to some plant hormones, in this case, *SlBES1* genes were generally induced by IAA while repressed by GR24. On the contrary, *SlBES1* genes could also be affected by some plant hormone with an opposite trend, for instance, ABA induced the expression of *SlBES1.6* and *SlBES1.8* while repressed *SlBES1.3* and *SlBES1.5*. Taken together, the variational expression of *SlBES1* genes under different plant hormone treatment implied that this gene family involved in multiple hormonal signals in a complicated way. The detailed role of this gene family in the crosstalk of plant hormones thus was worth to studying and may provide us the new insight in the field.

### Expression profiles of *SlBES1* genes in response to stresses

**Fig. 9:**
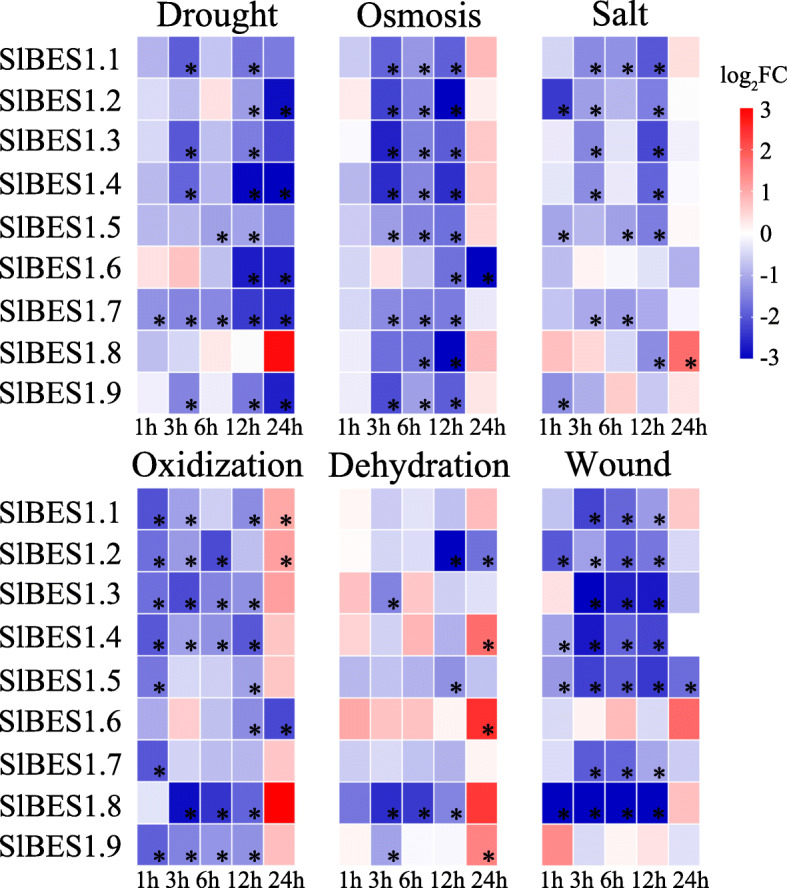
Expression profiles of *SlBES1* genes under stress treatments. Droughty (PEG6000), osmotic (Mannitol), oxidative (MV), salt (NaCl), dehydrated (Dehydration) and injured (Wound) stress were conducted to one-month-old tomato plants to analyze the responsiveness of *SlBES1* genes. Samples collected from those tomato plants well-watered were used as control. The relative expressions of *SlBES1* genes were detected by qRT-PCR after treated for 1 h, 3 h, 6 h, 12 h and 24 h. Data were converted to log_2_FC and visualized by heat map. Value for each time point represents the mean of three biological replicates. Red and blue colors indicate increased and decreased expression levels to the control respectively. * refer to significant differences with *p* < 0.05 compared to the corresponding mock controls

To further explore the potential responsiveness of *SlBES1* genes to biotic and abiotic stresses, we analyzed their expression profiles to drought, osmosis, salt, oxidization, dehydration and wound stress (Fig. 9). The presence of responsiveness to these stresses was summarized in Table S2 and marked as “Y”. Overall, *SlBES1* gene family could be affected by multiple stresses, which principally exhibited the downregulated trend in response to all of these six stresses. This indicated that *SlBES1* gene family may play the negative roles in tomato stress tolerance. In detail, four members (*SlBES1.2*, *SlBES1.3*, *SlBES1.4*, *SlBES1.5*) were hyperresponsive to all treatments analyzed here. Besides, at least four treatments can repress or induce the other five members. Notably, the strongest responsiveness of *SlBES1* family genes was detected after the wound treatment. In contrast, the relative mild responsiveness was observed in salt stress. The extensive involvement of *SlBES1* genes in response to these stresses implied the potential important functions of them.

## Discussion

*BES1* transcription factors were widely present in plants. Since its definition in *Arabidopsis*, the genome-wide identifications of *BES1* gene family had been conducted in many species, including *Brassica rapa* [45], *Brassica napus* [32], *Brassica rapa ssp. pekinensis* [46], *Zea mays* [47], Legume [31], *Gossypium* [29], *Glycine max* [48] and *Malus domestica* [49], while few reports focused on the functions of this gene family in tomato (*Solanum lycopersicum*). In our study, nine *BES1* genes were identified in tomato (Table 1), in which seven members were confirmed acting as TFs by combining the investigation of subcellular localization and transactivation activity whereas another two members were not (Figs. 5, 6). According to the conserved amino acid residues analysis, SlBES1 proteins possessed the same conserved N-terminal and serine-rich C-terminal (potentially responsible for DNA binding and phosphorylation respectively) in their BES1-type domain as observed in *Arabidopsis* (Fig. 1c). Besides, tomato *BES1* family shared very similar gene structure with their *Arabidopsis* homologs, in CDS region, the exon, BES1-type domain and EAR-motif showed highly identical in the number, location and length (Fig. 3b). Moreover, the conserved MEME motifs of BES1 proteins also exhibited corresponding permutation and combination with their phylogenetic relationship (Fig. 3c). These results implied the possibility that the *BES1* gene family may function conserved and redundant in tomato and *Arabidopsis*. Indeed, *AtBES1/AtBZR1* and *AtBEHs* exhibited functional redundancy in a certain extent, high ordered mutant of them resulted in the male sterility phenotype in *Arabidopsis* while single, double, triple, and quadruple mutants didn’t show such a phenotype [25, 26]. Under the guidance of these results, we could assume that multiple mutant of *SlBES1* genes may also influence the same biological processes.

As reported previously, BES1 proteins were phosphorylated by the core negative regulator BRASSINOSTEROID-INSENSITIVE 2 (BIN2) in the absence of BR, and the phosphorylated BES1 mainly distributed in the cytoplasm. In the presence of BR, the activity of BIN2 was inhibited, meanwhile the phosphorylated BES1 was dephosphorylated by PROTEIN PHOSPHATASE 2A (PP2A) and translocated subsequently from the cytoplasm to the nucleus [15, 50, 51]. Corresponding with this, the subcellular localization of AtBES1 showed the presence both in the nucleus and cytoplasm [13, 52]. Similarly, in our investigation, seven tomato BES1 members exhibited both nuclear and cytoplasmic localization (Fig. 5), which in turn suggested that tomato BES1 proteins also kept with the regulation of the phosphorylation. What’s more, another two special BES1 proteins, BMY2 and BMY4, were reported to locate in the nucleus and function as TFs in *Arabidopsis* [27]. Different with this result, their tomato homologs SlBES1.7 and SlBES1.1 didn’t localize to the nucleus (Fig. 5, S2), implying that functional differentiation of these two genes may occur. Given that four of nine *Arabidopsis* β-amylases located in the chloroplast [53], we investigated whether SlBES1.1 and SlBES1.7 possessed the same localization with them. While still, neither SlBES1.1-GFP nor SlBES1.7-GFP fluorescence overlapped with chlorophyll auto-fluorescence (Fig. S2), indicating the non-chloroplastic localization of SlBES1.1 and SlBES1.7. According to the size of fluorescence pigment, we supposed that these two genes may localize to endoplasmic reticulum. More experiments need to be performed to validate the subcellular localization of SlBES1.1 and SlBES1.7.

EAR motif was a well elucidated active repression motif in plant. EAR motif-containing proteins can act as transcription factors to directly repress downstream gene transcription by histone modifications, or otherwise, act as transcription regulators to regulate the activity of transcriptional activators by binding to them, thus decreased the expression level of target genes [34, 54]. By analyzing the gene structure and amino acid sequences, we found the presence of EAR motif in all those *BES1* genes annotated as TFs (Fig. 3b), which suggested the potential transcriptional repression function of these genes. Among them, however, five members (*SlBES1.3*, *SlBES1.4*, *SlBES1.5*, *SlBES1.6* and *SlBES1.9*) showed the transcriptional activation activity in yeast, and only two members (*SlBES1.2* and *SlBES1.8*) acted as transcriptional repressor (Fig. 6). In fact, it was reported that *AtBZR1* played dual roles in BR homeostasis and signaling despite the EAR motif present in its C-terminal. In this case, AtBZR1 can not only repress BR biosynthetic genes but activate downstream BR-responsive genes by directly binding to their promoters [14]. In a recent report published by Jia et al. [55], SlBZR1 (also named SlBES1.9 in our research) functioned as transcriptional repressor, which was opposite to our result. In their investigation, the *Arabidopsis* leaf protoplasts were used as the material to assess the transactivation ability of SlBZR1. The expression of reporter was not significantly changed by SlBZR1 in the absence of VP16, while in the presence of VP16, the transcriptional activation activity of VP16 was significantly reduced by SlBZR1. Given the different intracellular environment between yeast and *Arabidopsis* leaf protoplast, we may suppose that several different proteins may influence the transactivation activity of SlBZR1 by directly interaction or competition, resulting the presence of opposite transactivation ability. Similarly, AtBES1 also possessed the EAR motif in its C-terminal and functioned both as activator and repressor [7, 15]. This phenomenon was pervasive among transcription factors, for example, *Arabidopsis* Yin Yang 1 (AtYY1) contained both activation and repression domains, residues 51–193 in its N-terminal showed strong repressive activity while residues 256–387 in its C-terminal had more than 4-fold activating activity, resulting the full length of AtYY1 a weak transcriptional repression activity [56]. Thus we may assume that those *SlBES1* genes exhibited transcriptional activation activity in yeast could also contain an activation domain apart from the repressive EAR motif, making them can either activate or repress downstream genes under particular circumstances.

A tomato *BES1* gene, *SlBES1.8*, which was grouped in D in the NJ-tree (Fig. 2), had no closed homolog in *Arabidopsis*. Besides, it owned a simpler gene structure and only one conserved motif analyzed here (Fig. 3), implying the speciality of this gene. Subcellular localization showed that *SlBES1.8* localized in the nucleus and cytoplasm (Fig. 5), combined with the transcriptional repression activity concluded by the analysis in yeast and dual-luciferase assay (Fig. 6), we could regard it as a transcriptional repressor. Development- and tissue-specific expression pattern showed an exclusively relative high expression level of *SlBES1.8* in floral organ (Fig. 7), which provided the possibility that *SlBES1.8* may contribute to the fruit set and early development of tomato. Consistent with this assumption, *SlBES1.8* could be induced by both auxin and gibberellin (Fig. 8), two important plant hormones in regulation of fruit setting and development. What’s more, the auxin and gibberellin responsive *cis*-elements, AuxRR-core and TATC-box, were also observed in the promoter region of *SlBES1.8* (Fig. 4). Taken together, we could speculate that *SlBES1.8* may have important function in tomato fruit set and development, which was not reported in the function of other *BES1* members.

Among all plant hormone treatments, the most obvious change in *SlBES1* expression occurred when exposed to GR24, in which *SlBES1* genes were significantly repressed in early treatment stages (Fig. 8). It was reported that MORE AXILLARY GROWTH LOCUS 2 (MAX2), a critical strigolactone (SL) signaling component, can interact with BES1 and its homologs and regulate AtBES1 degradation, this process was dependent on the 26S proteasome and promoted by GR24 [57]. Furthermore, a putative SLs receptor, AtD14, can also enhance the degradation of AtBES1 [57]. Given that the decreased expression level of *SlBES1* genes (Fig. 8), we may assume that SlBES1 proteins were under the same degradation regulation in tomato. Beyond this, however, the transcription levels of *SlBES1* genes were not greatly influenced by the hormone treatments (such as ethephon) despite diacritical expression changes showed in some time points (Fig. 8). It was well elucidated that BES1 and BZR1 functioned in the dephosphorylated form, hormone treatments may not affect their mRNA levels but change their phosphorylation status. Indeed, the expression level of *AtBES1* was not affected by EBL treatment, whereas appeared to be stabilized in the dephosphorylated form [13]. In this study, EBL treatment also didn’t change the transcriptional levels of *SlBES1* genes in the early treatment stage, while some members, such as *SlBES1.6* and *SlBES1.2*, were induced or repressed after treated for 8 h (Fig. 8). Similar to EBL treatment, the expression levels of *AtBES1* and *AtBZR1* were also not influenced by GA_3_ treatment in *Arabidopsis*, while the dephosphorylated AtBZR1 appeared to accumulate after GA_3_ application [58]. In our investigation, most of *SlBES1* family genes had no responsiveness to GA_3_ treatment, except a slight induction of *SlBES1.6* and *SlBES1.8* and repression of *SlBES1.1* observed in some time points (Fig. 8, Table S1). AtBZR1 can interact with all DELLA proteins concluded by yeast two-hybrid. Besides, the physical protein-protein interaction of AtBZR1 with REPRESSOR OF ga1–3 (RGA), a DELLA family transcriptional regulator, was further validated by the colocalization, BiFC and co-IP assays. Moreover, AtBZR1 and RGA appeared to antagonize each other’s transcriptional activity [58]. Taken together, GA_3_ affected the function of *BES1* family primarily not in the transcriptional level but the translational level by regulating the BES1-DELLA interaction. Thus for the investigation of BES1 family mediated crosstalk with plant hormones, further studies should not only focus on the transcription regulation but also research the protein-protein interaction and phosphorylation status of BES1 family.

From the expression pattern in stress treatments, we can know that most of *SlBES1* genes were suppressed when exposed to the stresses and thus may function in a negative way in response to these stresses (Fig. 9). Many studies had proved this assumption, for example, AtBZR1 can directly repress the expression of *JUNGBRUNNEN1* (*JUB1*), a hydrogen peroxide-induced NAC transcription factor that functioned in promoting tolerance to various abiotic stresses [59, 60]. Besides, a previous study confirmed that BR signaling pathway can inhibit drought response by regulating the reciprocal inhibitory mechanism between BES1 and RESPONSIVE TO DESICCATION 26 (RD26), a negative regulator of the BR pathway. Moreover, RD26 can be induced by drought and promote the expression of drought-regulated genes thus enhance drought tolerance of plant. While BES1 can repress the expression of RD26 and other drought-related genes and lead to the inhibition of drought response [61]. Hence, we speculated that knock down of *SlBES1* gene family may improve the stress resistance and thus raise the yield of tomato.

Overall, this study provided valuable information for *SlBES1* family, including their genomic characterization, protein functional features and expression patterns in different tissues and in response to plant hormones and stresses. The results offered important clues for functional research of *SlBES1* genes and for the understanding of hormone signal crosstalk and stress resistance of tomato.

## Conclusions

In this study, nine *BES1* genes were identified in tomato. A systematic genome characterization was subsequently analyzed for this family, including chromosomal location, conserved amino acid residues within BES1-type domain, evolutional relationships, gene structures, conserved motifs and *cis*-elements. Subcellular localization and transactivation activity of *SlBES1* genes were further investigated. Besides, the expression profiles of *SlBES1* genes in different tissues showed potential important function in tomato fruit set and development. Moreover, the critical regulatory roles were implied by the expression patterns of *SlBES1* genes in response to plant hormones and stresses. Hence, our results lay a foundation for the functional research of *SlBES1* family.

## Methods

### Plant materials and growth conditions

Tomato plants (*Solanum lycopersicum* cv. Micro-Tom, a tomato dwarf cultivar obtained from Laboratory of Genomics and Biotechnology of Fruit, INRA, University of Toulouse, France) were transplanted on soil in greenhouse after germinated for 12 days on MS/2 medium. The suitable growth conditions were set to 16/8 h light/dark cycle, 25/20 °C day/night temperature and 60% relative humidity. Tobacco plants (*Nicotiana benthamiana* L.) were directly planted on soil under the same growth conditions. All plants were irrigated with nutrient solution once a week. Samples analyzed in development- and tissue-specific expression were collected from tomato seedling (12 DPG), one-month-old tomato (30 DPG) and other tomato tissues in corresponding developmental stages. Seedling, root, stem and leaf were collected from at least 8 independent healthy plants. Anthesis flower, flower at 2 days before anthesis and corresponding floral organ were collected from at least 20 independent healthy plants. Fruit samples at each stage were collected from at least 10 individual fruits. All samples were frozen immediately and mixed thoroughly after grinded. Each tissue group contained three independent biological samples and four technical repetitions for each sample were performed in qRT-PCR.

### Identification of *BES1* genes in tomato

To identify *BES1* gene family in tomato, the *AtBES1* was first obtained from The Arabidopsis Information Resource (TAIR) database (https://www.arabidopsis.org/). Full length of amino acid sequence of *AtBES1* was then used to search the BES1-type domain by CD-search in NCBI (https://www.ncbi.nlm.nih.gov/cdd/?term=). The amino acid sequence of BES1-type domain was used as a BLASTP query in Solanaceae Genomics Database (http://solgenomics.net/, Tomato Genome proteins, ITAG release 4.0) with an e-value of 10^− 10^. What’s more, Phytozome database (https://phytozome.jgi.doe.gov/pz/portal.html#!info?alias=Org_Slycopersicum) was also used to search *BES1* gene family in tomato. The presence of BES1-type domain in candidates obtained above were further confirmed by CD-search and SMART (http://smart.embl-heidelberg.de/). Taken together, candidates contained the BES1-type domain were regarded as *BES1* genes in tomato. *BES1* gene family in Arabidopsis (*A. thaliana*), pepper (*C. annuum*), soybean (*G. max*), rice (*O. sativa*) and maize (*Z. mays*) were collected from TAIR, Solanaceae Genomics Database, *Glycine max* Wm82.a2.v1 (https://phytozome.jgi.doe.gov/pz/portal.html#!info?alias=Org_Gmax), Rice Genome Annotation Project Database (http://rice.plantbiology.msu.edu/index.shtml) and *Zea mays* database (http://www.gramene.org/) respectively.

### Bioinformatic analyses of tomato *SlBES1* genes

The genomic loci of *SlBES1* genes were collected from Solanaceae Genomics Database. Besides, the molecular weight (MW) and isoelectric point (pI) of *SlBES1* genes were calculated by ProtParam tool in ExPASy Server (https://web.expasy.org/protparam/). ClustalX2.1 software [62] was used to conduct the multiple sequence alignment with full length sequences of nine SlBES1 proteins and eight AtBES1 proteins. The alignment result was further processed by ESPript 3.0 (http://espript.ibcp.fr/ESPript/cgi-bin/ESPript.cgi) to output the picture. For the conserved amino acid residues analysis of *SlBES1* genes, the BES1-type domain of each SlBES1 protein was confirmed by CD-search and subsequently visualized by WebLogo (http://weblogo.berkeley.edu/). What’s more, to analyze the evolutionary relationship, full length of BES1 proteins from tomato, *Arabidopsis*, pepper, soybean, rice and maize were aligned by MUSCLE program in MEGA X [63] with default settings. A Neighbor-joining Tree was then constructed based on the alignment result, and the Interactive Tree Of Life (iTOLv5) online tool (https://itol.embl.de/) was finally used to polish the NJ-tree. TBtools [64] and One Step MCScanX was used for gene synteny analysis among tomato, *Arabidopsis*, pepper and rice, and the result was further visualized by Multiple synteny Plot. Additionally, the structure of *BES1* genes was visualized by Gene Structure Display Server (GSDS 2.0) (http://gsds.cbi.pku.edu.cn/), and the conserved amino acids motifs of BES1 proteins were explored through MEME Suite (http://meme-suite.org/tools/meme). To explored the *cis*-elements in the promoter region of *SlBES1* genes, 2 kb sequences in the upstream of *SlBES1* coding sequences were used to submit into PlantCARE database (http://bioinformatics.psb.ugent.be/webtools/plantcare/html/).

### Subcellular localization of SlBES1 proteins

To determine the subcellular localization, full length of coding sequences without stop codon of *SlBES1* genes were fused into pCXDG-GFP vector. The fusion plasmids were subsequently transformed into *Agrobacterium tumefaciens* (GV3101). Leaf of one-month-old tobacco was used to transiently express the fusion SlBES1-GFP proteins. The green fluorescence was observed through the laser scanning confocal microscope (Leica TCS SP8, Germany) after infected for 3 days.

### Transactivation activity analysis in yeast

The open reading frames (ORFs) of *SlBES1* genes were amplified and ligated into pGBKT7-GAL4BD plasmid. The fusion GAL4BD-*SlBES1* constructs were further transformed into Y2H Gold yeast cells. The SD/−Trp medium plates were used to cultivate the yeast transformants. The α-galactosidase activity of the transformants was identified by X-α-gal and the expression of *AUR1-C* was screened by Aureobasidin A (AbA, Clontech, USA).

### Dual-luciferase assay

The ORFs of *SlBES1.2* and *SlBES1.8* were amplified and ligated into pEAQ-GAL4BD plasmid as the effector. Besides, VP16 was selected to constructed into pEAQ-GAL4BD as the positive control. The double-reporter vector, pGreenII 0800-LUC, which contained the GAL4-binding element (5 × GAL4) fused with the minimal TATA region of CaMV35S to drive the expression of firefly luciferase (LUC), was considered as the reporter. The renilla luciferase (REN) driven by CaMV35S was used as the internal control.

The effectors and reporter were transformed into *Agrobacterium tumefaciens* (GV3101) respectively, and co-infected the one-month-old tobacco leaf following the ratio of effector: reporter = 9: 1. The activity of LUC and REN were measured after co-infected for 3 days by the Dual-Luciferase Reporter Assay System (Promega, USA). At least six biological replicates were performed for each combination. Finally, the LUC/REN ratio was calculated to assess the transcriptional activation activity of SlBES1 proteins.

### Hormone and stress treatments

For hormone treatments, tomato seedlings were first germinated and grown on solid MS/2 medium. After germinated for 12 days, tomato seedlings were soaked into liquid MS/2 medium containing 20 μM IAA, 10 μM 6-BA, 20 μM GA_3_, 100 μM ABA, 20 μM Ethephon, 0.5 μM EBL, 20 μM SA, 50 μM MeJA and 5 μM GR24 respectively and incubated in the dark at 25 °C. Samples were collected after treated for 1 h, 2 h, 4 h, 8 h and 16 h respectively. Samples collected from those seedlings soaked into MS/2 medium without any hormone at each time point were used as control. Three individual seedlings were collected for one sample, and three samples were collected for each treatment at each time point.

One-month-old tomato plants were subjected to the stress treatments. Droughty, osmotic, oxidative and salt stress treatments were carried out by soaking the tomato plants into solutions containing 20% (m/v) PEG6000, 100 mM mannitol, 150 μM methyl viologen (MV) and 200 mM NaCl respectively followed by cultivating at standard conditions. For dehydrated stress treatment, tomato plants were removed the soil and cleaned by water, then placed on the filter papers and naturally dried at room temperature. For injured stress treatment, tomato leaves at the same position were pierced with tweezers. Tomato plants well-watered were used as control. Samples were collected after treated for 1 h, 3 h, 6 h, 12 h and 24 h. Leaves at the same position of three individual plants were harvested as one sample, and three samples were collected for each treatment at each time point.

### RNA isolation, cDNA synthesis and quantitative real-time PCR analysis

Total RNA was extracted with RNAprep Pure Plant Kit (Tiangen Biotech, China) according to the manufacturer’s instructions. The integrity of total RNA was detected by agarose gel electrophoresis and the concentration was measured by NanoDrop 1000 (Thermo, USA). The first strand cDNA was synthesized by PrimeScript™ RT reagent Kit with gDNA Eraser (Perfect Real Time) (Takara, Japan) with 2 μg total RNA for each 40 μL reaction. The cDNA products were diluted to 5-fold with deionized water before use. TB Green® *Premix Ex Taq*™ II (Tli RNaseH Plus) (Takara, Japan) was used to conduct qRT-PCR on the CFX96 Touch™ Real-Time PCR Detection System (BIO-RAD, USA). Two microliter diluted cDNA was used in each reaction, other reaction components and conditions were performed following the manufacturer’s instructions. The relative expression was calculated by the 2^-ΔΔCt^ method and visualized as heatmaps by TBtools.

## Supplementary Information

**Additional file 1: Figure S1.** The detailed sequence logos of those 10 conserved motifs in MEME analysis. **Figure S2.** Subcellular localization analysis of *SlBES1.1* and *SlBES1.7*. **Figure S3.** Relative expression of the reference genes under corresponding hormone treatments. **Table S1**. Summary of the responsiveness of *SlBES1* family to hormone treatments. **Table S2.** Summary of the responsiveness of *SlBES1* family to stress treatments. **Table S3**. Primers used in this study.

## Data Availability

The sequence information of tomato and *Arabidopsis BES1* family genes were collected from Solanaceae Genomics Network (SGN, SL4.0, https://solgenomics.net/) and The Arabidopsis Information Resource (https://www.arabidopsis.org/) respectively. The amino acid sequences of BES1 proteins in pepper, soybean, rice and maize were collected from SGN, soybean genome database (Wm82.a2.v1, http://phytozome.jgi.doe.gov/pz/portal.html), Rice Genome Annotation Project Database (http://rice.plantbiology.msu.edu/index.shtml) and *Zea mays* database (http://www.gramene.org/) respectively. *Cis*-elements were obtained from PlantCARE database (http://bioinformatics.psb.ugent.be/webtools/plantcare/html/). The *BES1* family expression data were generated by qRT-PCR and were available from the corresponding authors when needed. All other data supporting the results are included within the article and its Additional files.

## Abbreviations

AA: Amino acid
AbA: Aureobasidin A
BES1: BRI1-EMS-SUPPRESSOR 1
BZR1: BRASSINAZOLE-RESISTANT 1
CDS: Coding sequence
ChIP-chip: Chromatin immunoprecipitation-microarray
EAR: Ethylene-responsive element binding factor-associated amphiphilic repression
GFP: Green fluorescent protein
MW: Molecular Weight
ORFs: Open reading frames
pI: Theoretical Isoelectric Point
qRT-PCR: Quantitative real-time polymerase chain reaction
TFs: Transcription factors
UTR: Untranslated region
