# Supplementary material for: Genome-wide identification, characterization and expression analysis of BES1 gene family in tomato
Source: BMC Plant Biol. 2021 Mar 30;21:161. doi: 10.1186/s12870-021-02933-7 (PMC8010994; doi:10.1186/s12870-021-02933-7)
Supplement: Supplementary file 1 — Additional file 1: Figure S1. The detailed sequence logos of those 10 conserved motifs in MEME analysis. Figure S2. Subcellular localization analysis of SlBES1.1 and SlBES1.7. Figure S3. Relative expression of the reference genes under corresponding hormone treatments. Table S1. Summary of the responsiveness of SlBES1 family to hormone treatments. Table S2. Summary of the responsiveness of SlBES1 family to stress treatments. Table S3. Primers used in this study. [file 12870_2021_2933_MOESM1_ESM.docx]

**Genome-wide identification, characterization and expression analysis of *BES1* gene family in tomato**

Deding Su^1,2 †^, Wei Xiang^1,2 †^, Ling Wen^1,2^, Wang Lu^1,2^, Yuan Shi^1,2^, Yudong Liu^1,2 *^ and Zhengguo Li^1,2 *^

^1^ Key Laboratory of Plant Hormones and Development Regulation of Chongqing, School of Life Sciences, Chongqing University, 401331 Chongqing, China.

^2^ Center of Plant Functional Genomics, Institute of Advanced Interdisciplinary Studies, Chongqing University, 401331 Chongqing, China.

*** Correspondences:**

Yudong Liu, yudongliu@cqu.edu.cn;

Zhengguo Li, zhengguoli@cqu.edu.cn. Tel and Fax, +86-23-65678902.

**†** Deding Su and Wei Xiang contributed equally to this work.

**Table S1** Summary of the responsiveness of *SlBES1* family to hormone treatments

|  | IAA | 6-BA | GA3 | ABA | Ethephon | EBL | SA | MeJA | GR24 |
| --- | --- | --- | --- | --- | --- | --- | --- | --- | --- |
| SlBES1.1 | - | - | Y | Y | - | - | Y | Y | Y |
| SlBES1.2 | - | - | - | - | Y | Y | Y | - | - |
| SlBES1.3 | Y | - | - | - | - | Y | Y | Y | Y |
| SlBES1.4 | - | Y | - | - | - | Y | - | - | Y |
| SlBES1.5 | Y | - | - | Y | - | - | Y | Y | Y |
| SlBES1.6 | Y | Y | Y | Y | - | Y | Y | Y | Y |
| SlBES1.7 | - | - | - | - | - | Y | - | - | Y |
| SlBES1.8 | Y | - | Y | Y | - | Y | Y | Y | Y |
| SlBES1.9 | - | - | - | - | - | - | - | - | Y |
| Note: ‘Y’ indicates the presence of responsiveness, ‘-’ indicates no responsiveness. | | | | | | | | | |

**Table S2** Summary of the responsiveness of *SlBES1* family to stress treatments

|  | Drought | Osmosis | Salt | Oxidization | Dehydration | Wound |
| --- | --- | --- | --- | --- | --- | --- |
| SlBES1.1 | Y | Y | Y | Y | - | Y |
| SlBES1.2 | Y | Y | Y | Y | Y | Y |
| SlBES1.3 | Y | Y | Y | Y | Y | Y |
| SlBES1.4 | Y | Y | Y | Y | Y | Y |
| SlBES1.5 | Y | Y | Y | Y | Y | Y |
| SlBES1.6 | Y | Y | - | Y | Y | - |
| SlBES1.7 | Y | Y | Y | Y | - | Y |
| SlBES1.8 | - | Y | Y | Y | Y | Y |
| SlBES1.9 | Y | Y | Y | Y | Y | - |
| Note: ‘Y’ indicates the presence of responsiveness, ‘-’ indicates no responsiveness. | | | | | | |

**Table S3** Primers used in this study

| Gene | Primer | Sequence (5’-3’) | Purpose |
| --- | --- | --- | --- |
| Solyc01g094580 | qSlBES1.1-F | TTTGGCAAGGGAAGCAGGTT | qRT-PCR |
|  | qSlBES1.1-R | ACCCAGAAGGCACACCTTTC |  |
| Solyc02g063010 | qSlBES1.2-F | ACTGGCTTACGAACTCAAGGT |  |
|  | qSlBES1.2-R | GAGGCCTGTGTCCCTTGC |  |
| Solyc02g071990 | qSlBES1.3-F | AAGCTACCGAAGCACTGTGA |  |
|  | qSlBES1.3-R | CACCAGGGCTGAGTTGGTAA |  |
| Solyc03g005990 | qSlBES1.4-F | TAAACGGAGAGAACGACGGC |  |
|  | qSlBES1.4-R | CACATGTCCTACTGGCTTGC |  |
| Solyc04g079980 | qSlBES1.5-F | GTGGATTAAGAGCACAGGGGA |  |
|  | qSlBES1.5-R | TGGCTGAAGTGCCTCCAATC |  |
| Solyc07g062260 | qSlBES1.6-F | AAGCCGGATGGACAGTTGAG |  |
|  | qSlBES1.6-R | AAAGGAAGAGGAAGCAGGGC |  |
| Solyc08g005780 | qSlBES1.7-F | GCAAGCTGGTTGGACTGTTG |  |
|  | qSlBES1.7-R | GCCAGAAACTGGGCTCTCAA |  |
| Solyc10g076390 | qSlBES1.8-F | TCCTAAGCATGCAGACACCA |  |
|  | qSlBES1.8-R | TTGGCAAGTCCTTCACCGGA |  |
| Solyc12g089040 | qSlBES1.9-F | CTTATCGCAAGGGATGCAAGC |  |
|  | qSlBES1.9-R | CATGTTGGCATCAGCACGAG |  |
| Solyc04g081240 | qARF5-F | CTGAGATGGCCTAGTTCCAAGT |  |
|  | qARF5-R | CAGTTTGTGCTCCCAAAAAGGT |  |
| Solyc03g006880 | qGA20ox1-F | ACCATCTCGGGAATGTTTATCA |  |
|  | qGA20ox1-R | CTCCGGTTTCTGGCATGGT |  |
| Solyc04g008480 | qCLAU-F | ACCAACAAAAAGAGCTAGAATAGAG |  |
|  | qCLAU-R | TCATTGCTTGTTGTGAGAGGA |  |
| Solyc02g084850 | qTAS14-F | CTCAAGGCATGGGTACTGGT |  |
|  | qTAS14-R | CATCCTCCGACGAGCTAGAG |  |
| Solyc03g111720 | qE4-F | GACCACTCTAAATCGCCAGG |  |
|  | qE4-R | TTCCTGAGCGGTATTGCTTT |  |
| Solyc06g051750 | qCPD-F | CAAGGCCATTCAAGCGAGAAC |  |
|  | qCPD-R | GCAACAAGCAACGCCAGTAT |  |
| Solyc09g007010 | qPR1-F | GTGTCCGAGAGGCCAGACTA |  |
|  | qPR1-R | ATTGTTGCAACGAGCCCGA |  |
| Solyc01g079360 | qWRKY37-F | GCTCAGAAGGTTGTTATGACAGTGCA |  |
|  | qWRKY37-R | TTAGCTGAACAACCCTTTGAACTGC |  |
| Solyc09g065750 | qD27-F | ATCTAAGTGGGCGAGGGAGT |  |
|  | qD27-R | AGTGCACATTCCCACACAAT |  |
| Solyc01g056940 | qUBI-F | GCCGACTACAACATCCAGAAGG |  |
|  | qUBI-R | TGCAACACAGCGAGCTTAACC |  |
| Solyc01g094580 | GFP-SlBES1.1-F | ATGGCATCAGAGATGCAGAGATATG | Subcellular localization assay |
|  | GFP-SlBES1.1-R | CGATGGGAACTCAAGGACTGCTT |  |
| Solyc02g063010 | GFP-SlBES1.2-F | ATGACGGCCGGCACCGGCGGTGGAG |  |
|  | GFP-SlBES1.2-R | AGCACGTGCTTTTGCACTACCAAG |  |
| Solyc02g071990 | GFP-SlBES1.3-F | ATGACTTCCGGCACGAGGCTACCGA |  |
|  | GFP-SlBES1.3-R | TCTAGTGCTGGAGTTCCCAAGTGT |  |
| Solyc03g005990 | GFP-SlBES1.4-F | ATGACTTCCGGCACGAGGTTACCGA |  |
|  | GFP-SlBES1.4-R | TCTAGTGCTAGAGTTGCCAAGTGT |  |
| Solyc04g079980 | GFP-SlBES1.5-F | ATGATGTGGGAAGGTGGAGGGTTGC |  |
|  | GFP-SlBES1.5-R | CATCCGAGCAGTCCCACTTCCGA |  |
| Solyc07g062260 | GFP-SlBES1.6-F | ATGACGTCGGGAACAAGGATG |  |
|  | GFP-SlBES1.6-R | TCTTGTCTTTGAACTCCCAAGAGT |  |
| Solyc08g005780 | GFP-SlBES1.7-F | ATGAGCAATCCCCATCACCACATTTC |  |
|  | GFP-SlBES1.7-R | AGATTCCACATTGTTGATTTCTCC |  |
| Solyc10g076390 | GFP-SlBES1.8-F | ATGGGGGAAGATAAGAAAATTAGTG |  |
|  | GFP-SlBES1.8-R | AGAAGAGAGTGATAGAGTCAAAT |  |
| Solyc12g089040 | GFP-SlBES1.9-F | ATGATGTGGGAAGCTGGAGAATCAC |  |
|  | GFP-SlBES1.9-R | TATGCGAGCATTGCCACTTCCAAG |  |
| Solyc01g094580 | pGBKT7-SlBES1.1-F | ATGGCATCAGAGATGCAGAGATATGATA | Transcriptional activation activity assay |
|  | pGBKT7-SlBES1.1-R | CTACGATGGGAACTCAAGGACTGCTTCCC |  |
| Solyc02g063010 | pGBKT7-SlBES1.2-F | ATGACGGCCGGCACCGGCGGTGGAGGAT |  |
|  | pGBKT7-SlBES1.2-R | TTAAGCACGTGCTTTTGCACTACCAAGAG |  |
| Solyc02g071990 | pGBKT7-SlBES1.3-F | ATGACTTCCGGCACGAGGCTACCGA |  |
|  | pGBKT7-SlBES1.3-R | CTATCTAGTGCTGGAGTTCCCAAGTGTA |  |
| Solyc03g005990 | pGBKT7-SlBES1.4-F | ATGACTTCCGGCACGAGGTTACCGACAT |  |
|  | pGBKT7-SlBES1.4-R | CTATCTAGTGCTAGAGTTGCCAAGTGTAA |  |
| Solyc04g079980 | pGBKT7-SlBES1.5-F | ATGATGTGGGAAGGTGGAGGGTTGCCGG |  |
|  | pGBKT7-SlBES1.5-R | TCACATCCGAGCAGTCCCACTTCCGAGAG |  |
| Solyc07g062260 | pGBKT7-SlBES1.6-F | ATGACGTCGGGAACAAGGATG |  |
|  | pGBKT7-SlBES1.6-R | TTATCTTGTCTTTGAACTCCCAAGAGTAA |  |
| Solyc08g005780 | pGBKT7-SlBES1.7-F | ATGAGCAATCCCCATCACCACATTTCGA |  |
|  | pGBKT7-SlBES1.7-R | TTAAGATTCCACATTGTTGATTTCTCCGT |  |
| Solyc10g076390 | pGBKT7-SlBES1.8-F | ATGGGGGAAGATAAGAAAATTAGTGGTG |  |
|  | pGBKT7-SlBES1.8-R | TTAAGAAGAGAGTGATAGAGTCAAATTGA |  |
| Solyc12g089040 | pGBKT7-SlBES1.9-F | ATGATGTGGGAAGCTGGAGAATCACCAG |  |
|  | pGBKT7-SlBES1.9-R | TCATATGCGAGCATTGCCACTTCCAAGTG |  |
| Solyc02g063010 | pEAQ-SlBES1.2-F | ATGACGGCCGGCACCGGCGGTGGAGGAT | Dual-luciferase assay |
|  | pEAQ-SlBES1.2-R | TTAAGCACGTGCTTTTGCACTACCAAGAG |  |
| Solyc10g076390 | pEAQ-SlBES1.8-F | ATGGGGGAAGATAAGAAAATTAGTG |  |
|  | pEAQ-SlBES1.8-R | TTAAGAAGAGAGTGATAGAGTCAAA |  |

**
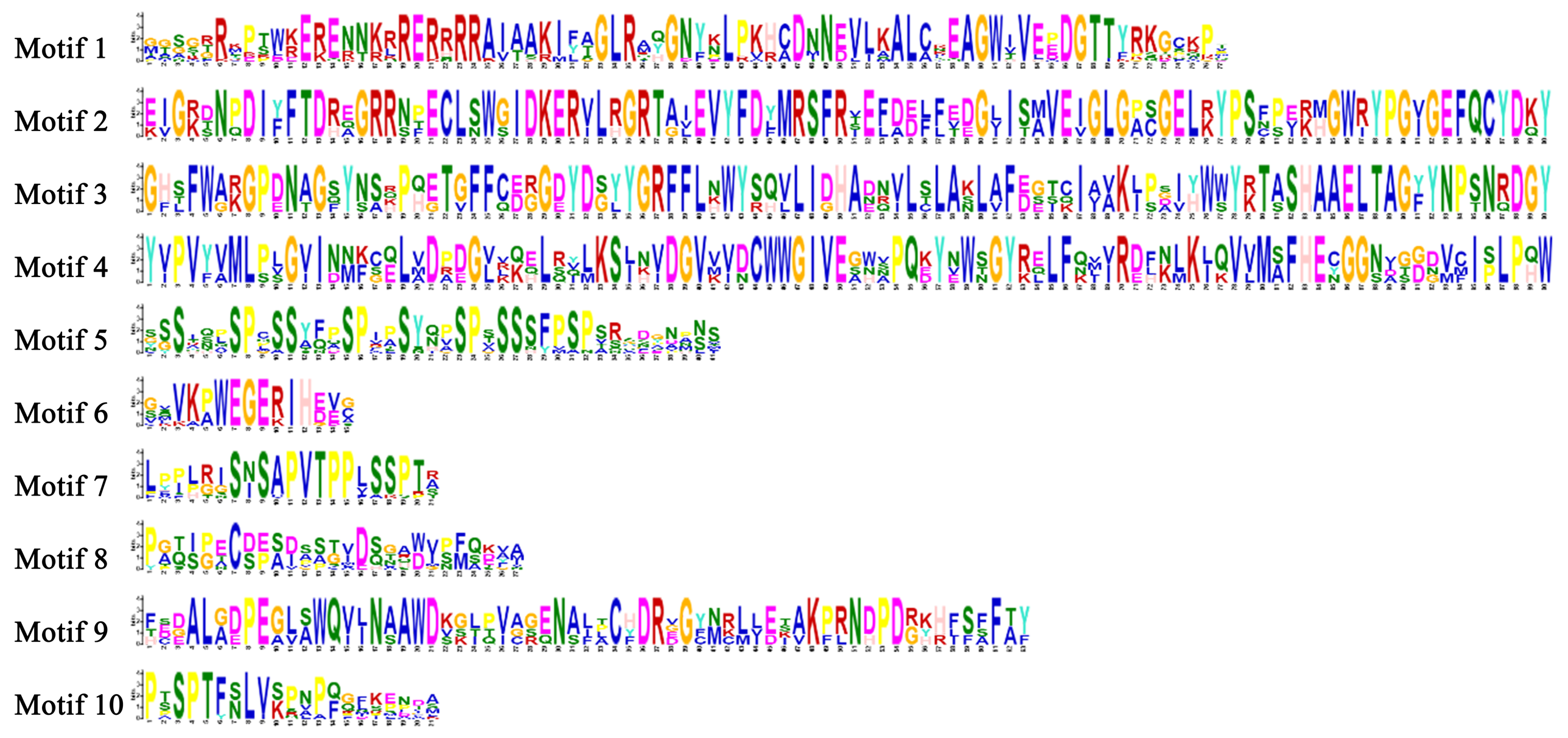
**

**Figure S1** The detailed sequence logos of those 10 conserved motifs in MEME analysis.


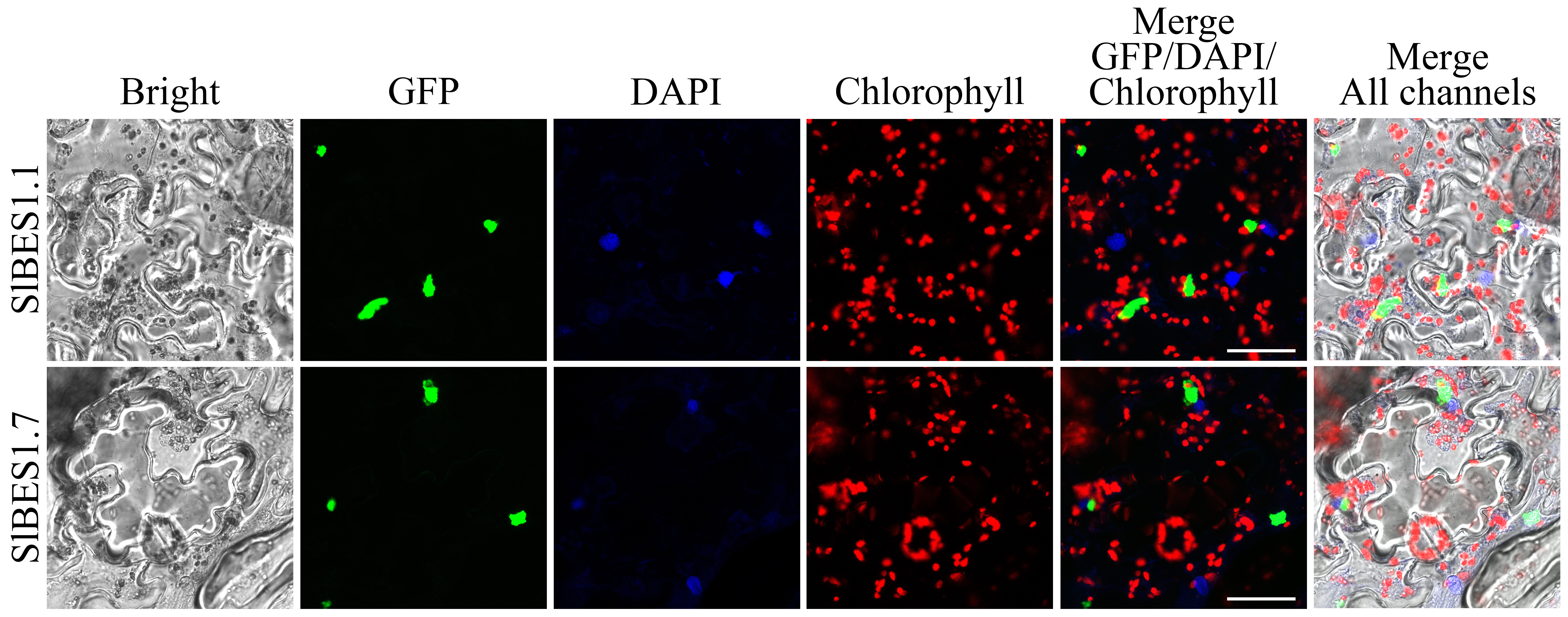


**Figure S2** Subcellular localization analysis of *SlBES1.1* and *SlBES1.7*. Tobacco leaves transiently expressed SlBES1.1-GFP and SlBES1.7-GFP fusion proteins were treated with DAPI followed by the observation through the laser scanning confocal microscope. Scale bars represent 50 μm.


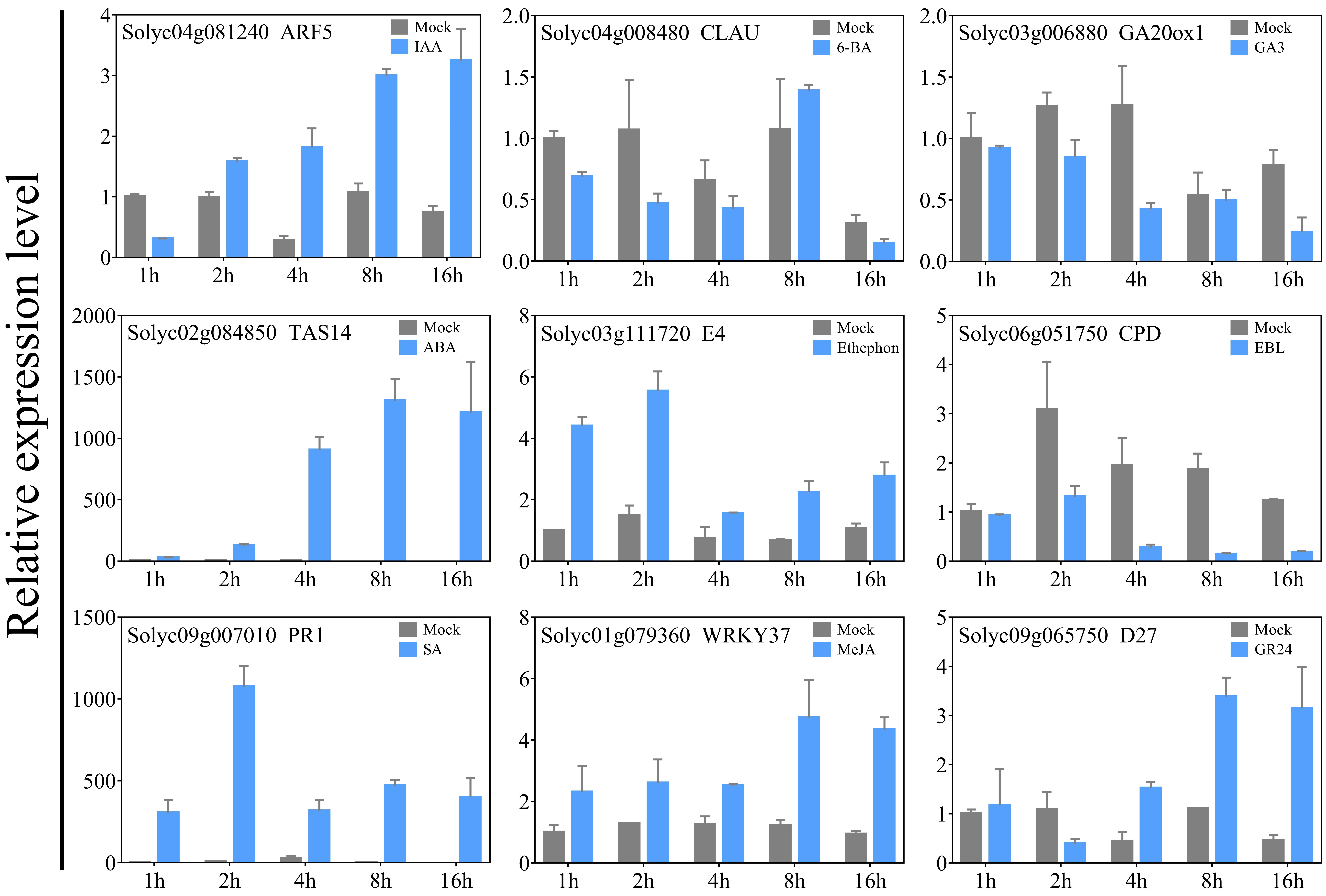


**Figure S3** Relative expression of the reference genes under corresponding hormone treatments. The relative expressions of reference genes were detected by qRT-PCR after treated for 1 h, 2 h, 4 h, 8 h and 16 h. Blue and gray columns represent the relative expression level of reference genes under hormone treatment and control respectively. Value of each column represents mean ± SE of three biological replicates.
